# Effect of the Strain Rate on the Damping and Mechanical Properties of a ZK60 Magnesium Alloy

**DOI:** 10.3390/ma13132969

**Published:** 2020-07-03

**Authors:** Xuhui Feng, Youping Sun, Yuwei Lu, Jiangmei He, Xiao Liu, Siyu Wan

**Affiliations:** 1 School of Mechanical and Transportation Engineering, Guangxi University of Science and Technology, Liuzhou 545006, China; fengxuhui1995@gmail.com (X.F.); jiangmeihe645@gmail.com (J.H.); wansiyu1996@gmail.com (S.W.); 2 Key Laboratory of High Temperature Wear Resistant Materials Preparation Technology of Hunan Province, Hunan University of Science and Technology, Xiangtan 411201, China; liuxiao0105@163.com

**Keywords:** high strain rate, ZK60 alloy, mechanical properties, damping capacity, damping peak

## Abstract

High strain rate rolling (HRSS) of a ZK60 magnesium alloy at 300 °C with a strain rate from 5 s^−1^ to 25 s^−1^ was used to research the effect of the rate on the mechanical properties and damping capacity of the ZK60 alloy. The results show that as the strain rate increases, the tensile strength decreases from 355 MPa at 25 s^−1^ to 310 MPa at 5 s^−1^. Two damping peaks (P1 and P2) are detected in the high strain rate rolled ZK60 alloys at different strain rates. The P1 peak appears at low temperatures and is caused by grain boundaries sliding. The P2 peak appears at high temperatures and is caused by recrystallization. As the strain rate increases from 5 to 20 s^−1^, the dynamic recrystallization (DRX) volume percent rises and the dislocation density decreases, both of which cause the P1 peak to become more and more obvious, and activation energy rises. At the same time, the dislocation density decreases and leads to a decrease in the storage energy, which reduces the recrystallization driving force and shifts the P2 peak to high temperatures. When the strain rate reaches 20 and 25 s^−1^, DRX occurs fully in the sheet, so the activation energy of the P1 peak and the temperature where the P2 peak appears are basically equal.

## 1. Introduction

Due to the rapid growth of the modern industry, machinery tends to have a high speed and be automated. However, the machines also possess many attributes that are unfavorable for the production of mechanical products, such as vibrating and producing noise [1]. These factors not only affect the accuracy and life of mechanical products but also increase energy losses and use of raw materials [2]. Failure analysis reports show that approximately two-thirds of industrial failures are caused by vibration and noise.

Magnesium alloys are the lightest structural material that also possess a high damping capacity, high specific strength and stiffness. Therefore, they are widely used in various fields [3,4,5]. However, magnesium alloys are limited in application because of their low mechanical properties [6]. In recent years, metal-based damping materials have been widely used to maintain structural stability over a wide range of temperatures and frequencies [7].

The production of high strength has been considered by alloying or plastic deformation. In recent years, the HSRR (high strain rate rolling) process is used to improve the mechanical properties of magnesium alloys with fine-grained microstructures and high-density dislocations [8,9,10,11].

The damping capacity of Mg alloys is caused by dislocation movement, which conforms with the G–L theory [12]. Therefore, to obtain a high-damping Mg alloy, the dislocations must be moved as much as possible. However, to achieve excellent mechanical properties in a material, the movement of the dislocations must be limited. The mismatch between the damping performance and the mechanical performance is common in Mg alloys [13]. Some studies have shown that magnesium alloys after plastic deformation do not fully conform to the G–L theory. They show unstable peaks in internal friction after plastic deformation [3,13,14,15,16], which may be due to other mechanisms that occur during the deformation of Mg alloys. Hence, the production of high strength and damping properties in magnesium alloys has been considered by rolling them.

This paper uses the HSRR process at different strain rates. After rolling, the changes in the grain size, twinning, mechanical properties and internal friction peaks of the rolled ZK60 alloy are studied.

## 2. Experimental Methods

A commercial semi-continuous ZK60 magnesium alloy ingot was used as materials for this experiment. The ZK60 magnesium alloy was first machined to an initial size with a length of 50 mm, a width of 50 mm and a thickness of 10 mm. After a two-step homogenization treatment at 330 °C for 24 h and then 420 °C for 4 h, the billets were preheated to 300 °C for 13 min before the HSRR process. The preheated billets were rolled by a two-roll mill with a roll diameter of 210 mm, and the effective strain rates were approximately 5, 10, 20 and 25 s^−1^. A reduction from 10 to 2 mm occurred with a single pass and was followed by water quenching immediately after rolling to retain the deformed grain structure, the HSRR process is displayed in Table 1.

**Figure 1:**
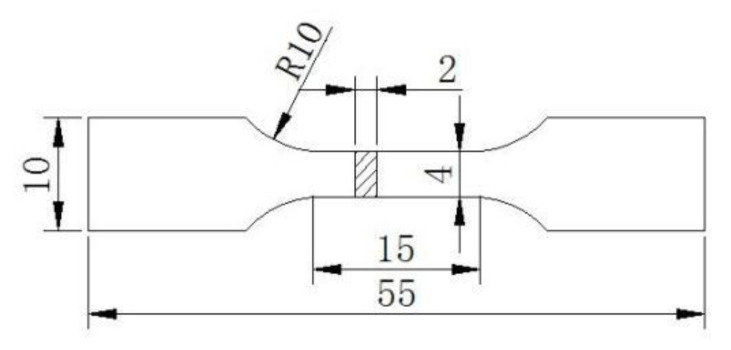
Tensile specimen size (mm).

The damping specimens were machined to size of 65 × 3 × 1.5 mm along the rolling direction (RD). The damping capacity was measured by a multifunctional internal friction analyzer (MFP-1000, institute of Solid State Physics, Chinese Academy of Sciences, Hefei, China). The experimental parameters were as follows: the strain amplitude was 4 × 10^−5^, the frequencies were 0.5, 1.0, 5.0 and 8.0 Hz, the temperature range was approximately from 30 to 400 °C, with 5 °C/min. The tensile test results are the average values of three measurements and were carried out with an electronic universal testing machine ETM105D along the RD (rolling direction) at a tensile rate of 2 mm/min at room temperature. The tensile specimen size is shown in Figure 1.

## 3. Results and Discussion

### 3.1. Effect of Homogenization on ZK60 Magnesium Alloy

**Figure 2:**
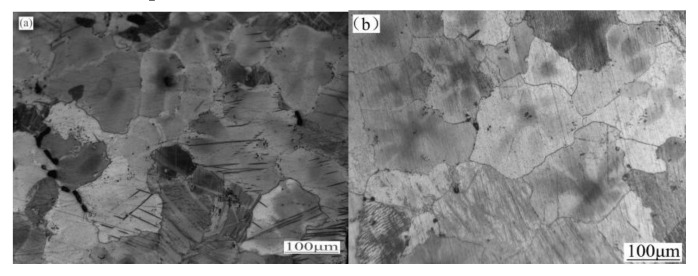
Optical microstructures of ZK60 magnesium alloy (**a**) as-cast (**b**) as-homogenization.

Figure 2a displays the microstructure of as-cast ZK60 magnesium alloy. It was observed that the average grain size was 98 µm, the second phase was distributed discontinuously along the grain boundaries and a few twins were distributed in the grains. Figure 2b displays the microstructure of the as-homogenization ZK60 magnesium alloy, after homogenization, the average grain size grew to 127 µm and only a few second phases were detected.

### 3.2. Microstructure of the Alloy after HSRR 

**Figure 3:**
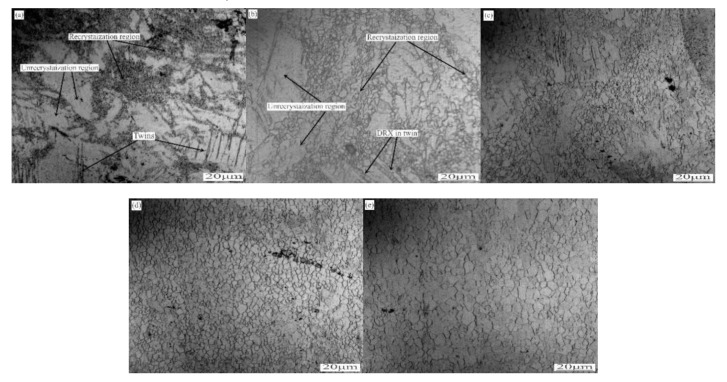
Optical images of the ZK60 magnesium alloy after HSRR under different strain rates. (**a**) 5 s^−1^ (**b**) 10 s^−1^ (**c**) 15 s^−1^ (**d**) 20 s^−1^ (**e**) 25 s^−1^.

Figure 3 shows the microstructure of the rolled ZK60 magnesium alloy. When the strain rate was low (5, 10, and 15 s^−1^), both twins and unrecrystallized regions were observed, and fine grains were formed in some of the twin lamellae and at the grain boundaries. These grains were smaller than the original grains, showing that these grains were formed by dynamic recrystallization (DRX). As the strain rate increased, the DRX volume percent rose. When the strain rate was 20 s^−1^, twins and “island” regions disappeared because DRX occurred fully in the sheet.

**Figure 4:**
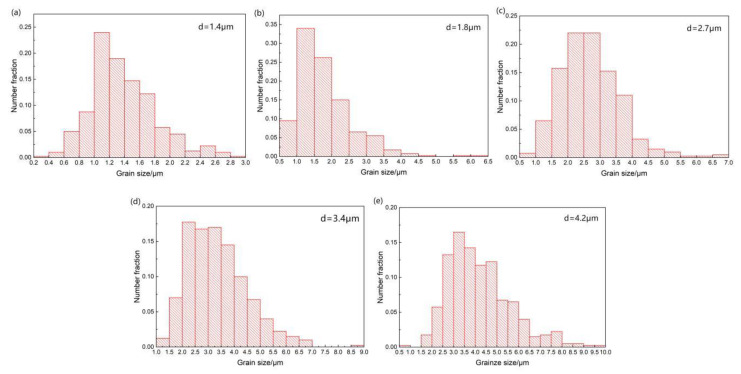
Grain size distribution of rolled ZK60 magnesium sheet under different strain rates. (**a**) 5 s^−1^ (**b**) 10 s^−1^ (**c**) 15 s^−1^ (**d**) 20 s^−1^ (**e**) 25 s^−1^.

Figure 4 displays the grain size distribution of the rolled ZK60 magnesium sheet under different strain rates. As the strain rate increased from 5 to 25 s^−1^, the grain grew. The grain sizes became 1.4, 1.8, 2.7, 3.4 and 4.2 µm.

The grains grew because a lot of deformation heat was generated by the HSRR. The contact time was short, so the heat could not be dissipated, the temperature of the whole sample rose, and the temperature rose as the strain rate increased [17]. As the temperature rose, the nucleation of the DRX grains and the migration of the grain boundaries were accelerated. Hence, the HSRR process accelerated the DRX process, and the twinned grains lost their original appearance. The uniformity of the structure increased, but the rising temperature also caused the grains to grow [11].

**Figure 5:**
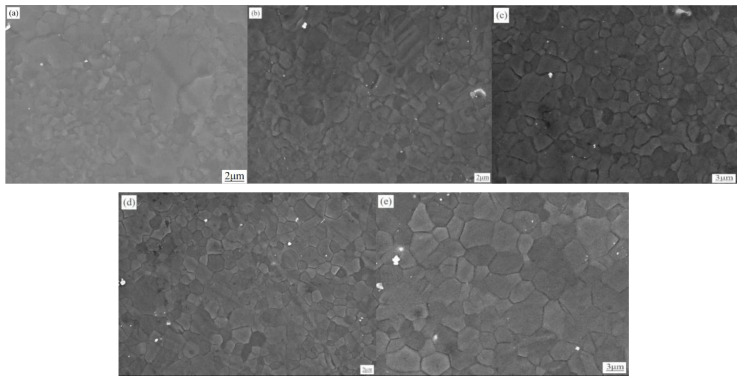
SEM images of the ZK60 magnesium alloy after HSRR under different strain rates. (**a**) 5 s^−1^ (**b**) 10 s^−1^ (**c**) 15 s^−1^ (**d**) 20 s^−1^ (**e**) 25 s^−1^.

**Figure 6:**
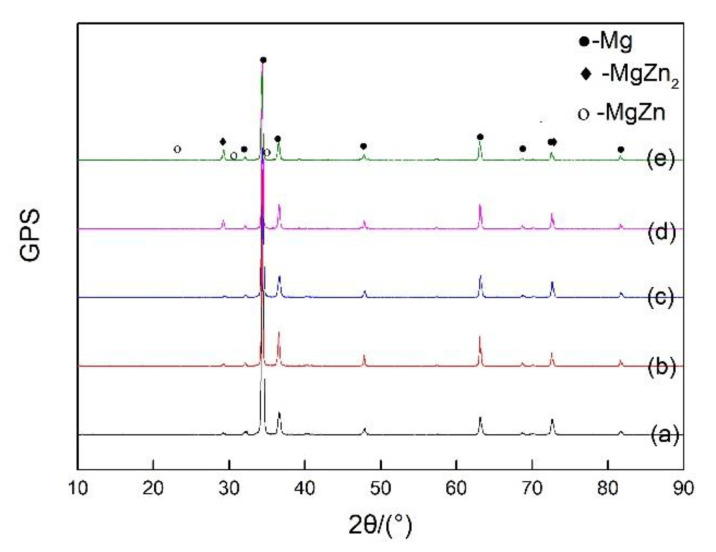
XRD pattern of the HSRR ZK60 magnesium alloy under different strain rates (**a**) 5 s^−1^ (**b**) 10 s^−1^ (**c)** 15 s^−1^ (**d**) 20 s^−1^ (**e**) 25 s^−1^.

Figure 5 displays the distribution of the precipitates after rolling. Figure 5 shows that only a few second phases in the rolled ZK60 sheet were found on boundaries and in the grains. Therefore, the strain rate had almost no effect on the second phase. The sample was analyzed by XRD to estimate the second phase. The XRD patterns of the HSRR ZK60 alloy are shown in Figure 6, which indicated that the precipitates in the alloys at various strain rates mainly consisted of the MgZn_2_ and MgZn phases.

### 3.3. Tensile Properties of the Alloy after HSRR

**Figure 7:**
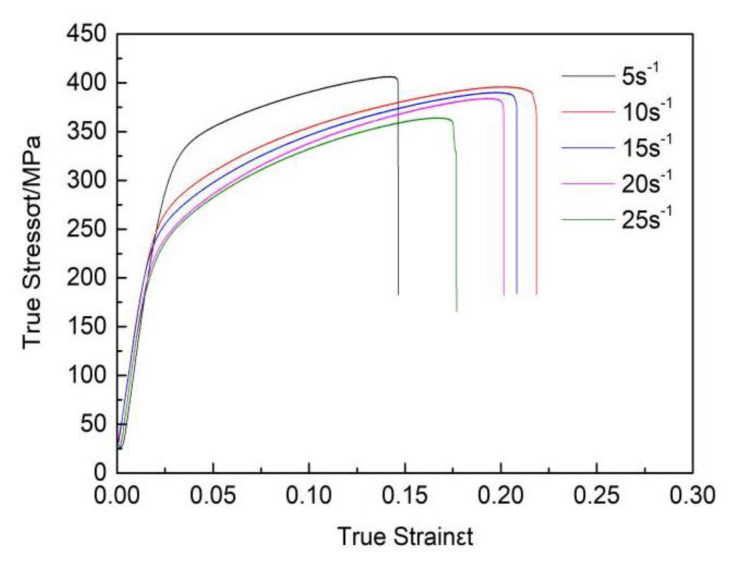
True strain-true stress curve for rolled sheets under different strain rates.

The true strain-true stress curves for the rolled sheets are displayed in Figure 7, and the tensile properties are shown in Table 2. All the high strain rate rolled sheets presented an excellent strength–ductility balance. As the strain rate increased, the tensile strength reduced from 355 MPa at 5 s^−1^ to 310 MPa at 25 s^−1^.

Combined with Figure 1, it can be found that when the strain rate was 5 s^−1^, the degree of recrystallization was lower than other strain rates. Studies [10,11] have shown that unrecrystallized regions have a high density of dislocation grids and dynamically recrystallized grains have relatively low densities; the reduction in the dislocation density then leads to a decrease in the mechanical properties. Additionally, the grain size at 5 s^−1^ is smaller than other strain rates, grain refinement helps increase tensile strength [9,18,19], therefore, it has the highest tensile strength at 5 s^−1^.

### 3.4. Damping Capacity of the Alloy after HSRR 

**Figure 8:**
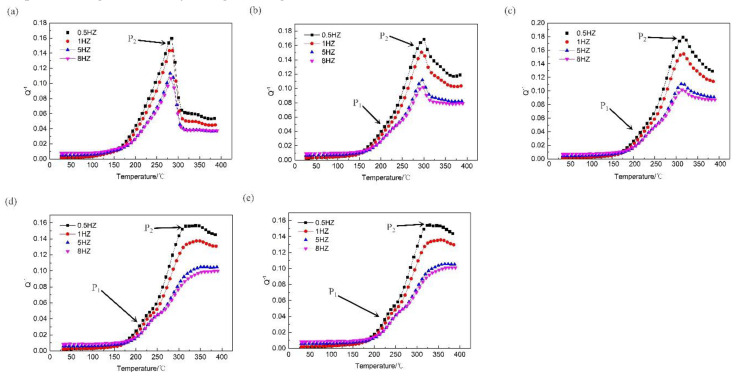
The temperature-dependent damping capacities with various frequencies under different strain rates. (**a**) 5 s^−1^ (**b**) 10 s^−1^ (**c**) 15 s^−1^ (**d**) 20 s^−1^ (**e**) 25 s^−1^.

Figure 8 shows the temperature-dependent damping capacity of the high strain rate rolled ZK60 alloy at different frequencies under different strain rates. Two peaks were observed at each rate, which are referred to as P1 (at low temperatures) and P2 (at high temperatures).

**Figure 9:**
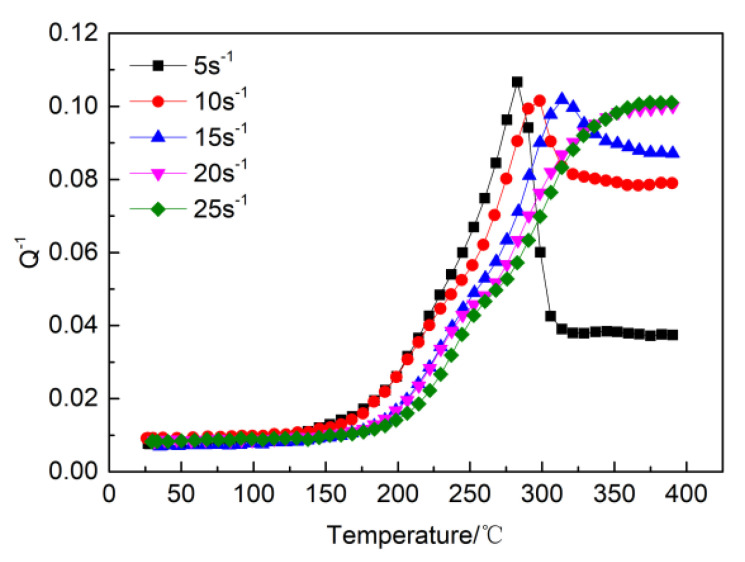
Temperature-dependent damping capacities with f = 1 HZ under different strain rates.

To further explain the effect of the strain rate on peak P1, the curve for the temperature-related damping of the high strain rate rolled ZK60 alloys with different strain rates was obtained. The measured data are shown in Figure 9 when the strain was 4 × 10^−5^ and frequency was 1 Hz. The P1 peak appeared at approximately 420 K, which is the same phenomenon observed by many researchers, and the experimental evidence showed that the P1 peak was caused by grain boundary sliding [3,13,20]. As the strain rate increased, the P1 peak became increasingly intense, but when the strain rate was 5 s^−1^, the P1 peak was not visible. When considering the results in Figure 1 (metallographic diagram), it can be concluded that as the strain rate increases, the DRX volume percent rises, thus, the volume fraction of DRX has a crucial effect on the P1 peak. At decreased strain rates, the DRX volume percent is low, so the dislocation density is high [10,11], leading to the P1 peak being masked by a high background from the internal friction [21].

As the frequencies increased, the P1 peak temperature shifted to an increased temperature, which implies that the appearance of the P1 peak is a thermally activated relaxation process [22]. The thermal activation could be explained by the Arrhenius equation, which is shown in formula (1) [23]:

(1) $$\tau =\tau_{0}e^{H/KT}$$

where τ is relaxation time, τ_0_ is exponent factor, H is activation energy, K is the Boltzmann constant and T is the peak temperature. Relaxation time τ affected the position of the internal friction peak, and its peak appeared at τω = 1 and ω = 2πf.

A logarithmic transformation was applied to Equation (2):

(2) $$\ln2\pi f+\ln\tau_{0}+\frac{H}{1000K}\times \frac{1000}{T}=0.$$

It is shown that *T* was related to the testing frequency, and *H* could be calculated by the slope of ln2π*f*~1000/*T*.

**Figure 10:**
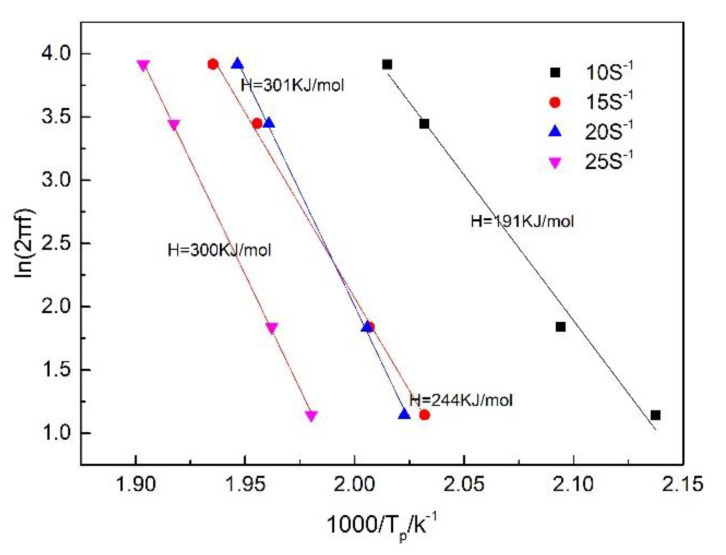
Arrhenius plots of ln(2πf) and 1000/Tp for the P1 peaks of rolled ZK60 magnesium alloy under different rates.

Figure 10 shows the Arrhenius plots of the internal friction peaks from the high strain rate rolled ZK60 alloy under different strain rates. The action energy H was calculated by the slope. As the strain rate increased, the activation energies were 191, 244, 301 and 300 kJ/mol. When considering the results in Figure 1 (metallographic diagram), we can conclude that DRX volume percent has a crucial influence on the activation energies. At decreased strain rates, there are many unrecrystallized regions and a high dislocation density, the dislocation walls formed by dislocation entanglement divide the original grains into many subgrains, and the subgrains should absorb dislocations into the lattice to further form low-angle and high-angle grain boundaries. Therefore, at a low activation energy, grain boundary slip can occur and generate a damping peak. As the strain rates increase, DRX volume percent rises, so the dislocation density decreases; therefore, the activation energy of the P1 peak increases with increasing rate, and grain boundary slip does not occur readily. Since DRX fully occurs in the sheet when the strain rates reach 20 s^−1^ and 25 s^−1^, the activation energy of the grain boundary slip peaks at these two rates are basically the same [24]. At the same time, by comparing the results for the 20 s^−1^ and 25 s^1^ rates again, it can be obtained that the grain size has little effect on the activation energy.

According to Figure 6, the height of the P2 peak increased with decreasing frequency, but the peak did not shift with a change in the frequency. This result implies that the P2 peak is not the result of a thermal relaxation process. 

Wang Chicun found that heat treatment can shift the P2 peak to increased temperatures. After sufficient annealing, the P2 peak disappears [24]. Zhou Hai found that after a second heating step, the P2 peak disappears due to the disappearance of twins [13]. Therefore, the P2 peak is considered as a recrystallization peak. To further explain the influence of strain rates on the P2 peak, the curve for the temperature-related damping of the high strain rate rolled ZK60 alloys with different strain rates was obtained. The measured data are shown in Figure 7 which were measured at a strain of 4 × 10^−5^ and frequency of 1 Hz. As the strain rates increased, the temperature at which the P2 peak appears became higher. Along with the data in Figure 1 (metallographic diagram), as the strain rate increased, unrecrystallized volume percent reduced. This led to a decrease in the storage energy of the ZK60 magnesium alloy and a decrease in the recrystallization driving force, which increased the recrystallization temperature.

It can be obtained from Figure 6 that the damping increased as the frequencies increased at low temperatures, but at high temperatures, the damping decreased as the frequencies increased.

At low temperatures, the dislocations played a main role in the damping mechanism. According to the K–G–L theory, in the low temperature, the damping due to dislocations can be expressed by the following formula [12,25]:

(3) $$Q^{-1}=Q_{a}^{-1}+Q_{f}^{-1}$$

(4) $$Q_{a}^{-1}=C_{1}\frac{\rho b^{2}}{\varepsilon_{0}}\exp(-\frac{C_{2}}{\varepsilon_{0}})$$

(5) $${Q_{f}}^{-1}=C_{3}\rho f^{2}/b^{2}$$

where *ρ* and *ε*_0_ are the dislocation density and strain amplitude, respectively; *b* and *f* are the vector and vibration frequency, respectively; and C_1_, C_2_ and C_3_ are physical constants.

In the experiments herein, the experimental amplitude was fixed (*ε*_0_ = 4 × 10^−5^), and the *ρ* and *b* of the same material with the same HSRR process were uniform. According to equation 4, *Qa* was unchanged under the same process, and according to equation 5, *Q_f_* increased as *f* increased. Therefore, at the same strain rate, the damping performance at room temperature increased with the frequency. 

At high temperatures, the main damping mechanism is due to grain interface sliding [3,20,26]. Interface sliding can only be activated at low frequencies. Therefore, the damping decreases as the frequency increases at high temperatures.

Grain boundaries provide viscosity in polycrystalline metals, and the resulting viscous flow at the grain boundaries produces thermal energy [27]. The dissipation of this thermal energy leads to an increase in the damping. As the volume fraction of DRX increases, the grain boundaries become relatively prevalent. Therefore, as the strain rate increases, the damping capacity at high temperatures increases.

## 4. Conclusions

As the strain rate increases, the tensile strength reduces from 355 MPa at 5 s^−1^ to 310 MPa at 25 s^−1^. When the strain rate is 5 s^−1^, because the DRX volume percent is less than other strain rates, the grain size is smaller than other strain rates, resulting in the maximum tensile strength.

Two damping peaks (P1 and P2) are detected in high strain rate rolled ZK60 alloys under different strain rates. The P1 peak appears at low temperatures and is caused by the grain boundaries sliding. The P2 peak appears at high temperatures and is considered a recrystallization peak. As the strain rate increases from 5 to 20 s^−1^, the DRX volume percent increases and the dislocation density decreases, resulting in the P1 peak increasing in intensity and an increase in the activation energy. At the same time, the dislocation density decreases, leading to a decrease in storage energy, which reduces the recrystallization driving force and shifts the P2 peak to higher temperatures. When the strain rate reaches and 25 s^−1^, complete recrystallization occurs, the activation energy of the P1 peak and the temperature at which the P2 peak appears are basically equal.

For the high strain rate rolled ZK60 alloy, dislocations play a dominant role in the damping mechanism at low temperatures, so the damping performance increases with increasing frequency. At high temperatures, grain boundary sliding becomes the dominant mechanism, so the damping performance decreases with increasing frequency. As the strain rate increases, the DRX volume percent rises and the number of grain boundaries increases. Hence, as the rate increases, the damping capacity increases at high temperatures. 

## Figures and Tables

**Table 1:** The HSRR process at different strain rates.

Rolling Temperature (°C)	Rolling Thickness (mm)	Wheel Speed (mm/s)	Strain Rate (s^−1^)
300	10–2	256	5
		512	10
		768	15
		1024	20
		1280	25

**Table 2:** Tensile properties for the rolled sheets with different strain rates.

Strain Rate (s^−1^)	Tensile Strength (MPa)	Elongation (%)
5	355	12.4
10	329	20.7
15	324	17.3
20	319	16.8
25	310	15.1
